# The Role of Respiratory Flora in the Pathogenesis of Chronic Respiratory Diseases

**DOI:** 10.1155/2021/6431862

**Published:** 2021-08-14

**Authors:** Mei-Ying Guo, Hao-Kun Chen, Hua-Zhong Ying, Fen-Sheng Qiu, Jun-Qi Wu

**Affiliations:** ^1^Zhejiang Key Laboratory of Experimental Animal and Safety Evaluation, Hangzhou Medical College, Hangzhou 310013, China; ^2^Clinical Laboratory, Jinhua Municipal Central Hospital Medical Group, Jinhua 321000, China

## Abstract

Large quantities of bacteria, including *Firmicutes*, *Actinobacteria*, and *Bacteroidetes*, colonize the surface of the respiratory mucosa of healthy people. They interact and coexist with the local mucosal immune system of the human airway, maintaining the immune stability and balance of the respiratory system. While suffering from chronic respiratory diseases, the microbial population in the airway changes and the proportion of *Proteobacteria* is increased in patients with asthma. The abundance of the microbial population in patients with chronic obstructive pulmonary disease (COPD) is decreased, and conversely, the proportion of *Firmicutes* and *Proteobacteria* increased. The diversity of airway microorganisms in cystic fibrosis (CF) patients is decreased, while pathogenic bacteria and conditional pathogenic bacteria are proliferated in large numbers. The proportion of *Firmicutes* and *Proteobacteria* is increased in patients with upper airway cough syndrome (UACS), which replaces the dominance of *Streptococcus* and *Neisseria* in the pharynx of a normal population. Therefore, a clear understanding of the immune process of the airway flora and the immune dysfunction of the flora on the pathogenesis of chronic respiratory diseases can provide new ideas for the prevention and treatment of human respiratory diseases.

## 1. Introduction

**Figure 1:**
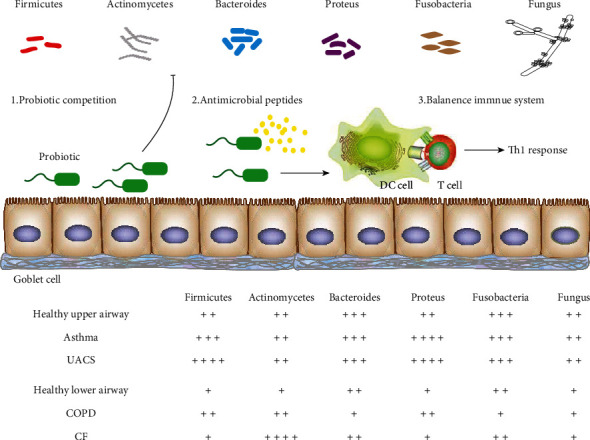
The profiles of respiratory flora in the occurrence and development of chronic respiratory diseases.

The human body carries a huge group of microbial populations, which constitutes a complex and delicate ecosystem. The complex interaction between microbes and the human immune system determines the health of the human body. The airway is an open cavity connecting the human body with the outside world. It is always invaded by external microbes, so it has strong local immunity. For example, the airway contains not only motile cilia, strong secretory goblet cells, and secretory IgA on the mucosal surface but also abundant mucosal-associated lymphoid tissue under the mucosa, which is sufficient to cope with the invasion of external pathogens [1]. Once the local immunity of the airway is disturbed, acute and chronic respiratory diseases such as asthma, chronic obstructive pulmonary disease (COPD), cystic fibrosis (CF), and upper airway cough syndrome (UACS) are likely to be triggered (Figure 1). For a long time, the airway, especially the lower airway, has been considered amicrobic [2, 3]. The airway is a dynamic ecosystem full of microbial populations, which is closely related to the immunity and inflammatory response of the host [4]. As is in the intestine, there are certain types and quantities of microbial populations in the airway of healthy people, that is, normal flora. Under the influence of external factors, these normal florae continuously evolve to maintain the dynamic balance of respiratory microecology and resist and avoid the invasion and colonization of pathogens to the airway [5, 6]. However, under the influence of external factors, such as environmental pollution and antibiotic abuse, changes in respiratory flora may result in pathogenic infection. Even some strains in the normal flora may turn into conditional pathogenic bacteria, causing several respiratory diseases. The stability of the airway flora is greatly affected by the external environment, which affects the local airway immune balance and even leads to the immune dysfunction of the respiratory system, thereby adding lots of difficulties to the clinical treatment of respiratory diseases. From the microecological perspective, it is of great significance to pay attention to the local immunity of the airway in the prevention and treatment of respiratory diseases [7, 8]. In recent years, with the development and application of molecular biology techniques, the research on the relationship between respiratory microflora with respiratory system immunity and diseases has developed rapidly. Growing studies have interpreted the role of complex respiratory microflora in the process of immune dysfunction in various respiratory diseases, which provides new ideas for exploring the pathogenesis and treatment of respiratory diseases [9, 10].

## 2. Flora Distribution and Local Immunity of the Airway in Healthy People

Large quantities of bacteria colonize the surface of the respiratory mucosa of healthy people, of which the main groups are *Firmicutes*, *Actinobacteria*, *Bacteroidetes*, *Proteobacteria*, and *Fusobacteria* [11]. Bacteria regulate the species and quantity through the quorum-sensing system and locally produced antimicrobial peptides to maintain dynamic balance and achieve peaceful coexistence with the human body. The floras in the upper and lower airways are highly homologous, and the quantity of upper airway flora is greater than that of the lower airway, which means that there are no specific microbes in the upper and lower airways [12]. This is different from the normal intestinal flora. The human airway is mainly defended by the local mucosal immune system. Mucosal epithelial cells act as a physical barrier separating the internal and external environments. They are the main structure of the local innate immune system and the main carrier of the airway flora. There are cilia on the surface of respiratory mucosal epithelial cells, which can transport bacteria of the lower airway upwards, while the mucus secreted by goblet cells and secretory IgA antibodies produced by submucosal lymphohistiocytes can further maintain the local chemical immune barrier function of the mucosa and prevent the invasion and colonization of pathogens [13, 14]. However, the local immune system of the airway neither equips with the strong acid and alkali on the surface of alimentary canal mucosa and the powerful germicidal mechanism of excellent enzyme system nor the extremely anaerobic environment, which determines that there is no significant difference in the species but only in the number of bacteria in the upper and lower airway. This is the evolutionary result of the interaction and symbiosis between the airway local immunity and the flora [15].

## 3. Immunity of Respiratory Flora and Asthma

**Figure 2:**
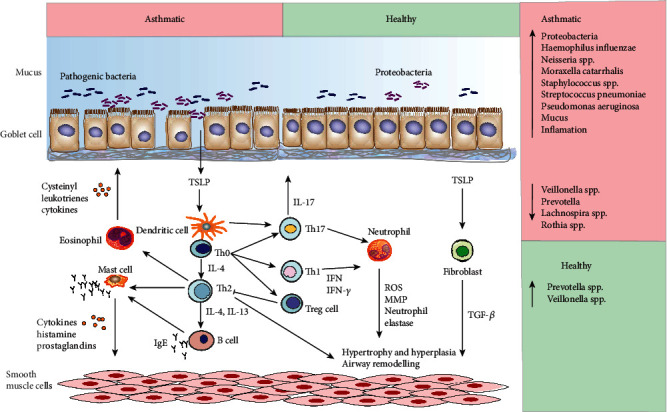
The roles of pathogens in the occurrence and development of asthma.

Asthma is a kind of disease that is well-recognized as immune dysfunction of the respiratory system, which is defined as a clinical syndrome of intermittent respiratory symptoms caused by viral upper respiratory infections, environmental allergens, or other stimuli, characterized by nonspecific bronchial hyperresponsiveness and airway inflammation [16, 17]. Due to the low positive rate of colony culture in sputum in the past and the insignificant effect of antibiotic treatment in the early stage of asthma, it was believed that there was no relationship between bacteria and asthma attacks. An epidemiological study had disclosed that bronchial infection could be the basis of asthma attacks in the adult during the outbreaks of bronchitis and pneumonia. This was also in line with the previously reported efficacy of macrolide antibiotics in the treatment of patients with chronic infectious asthma [18]. With the application of serological testing and PCR-based research methods, it had found that *Streptococcus pneumoniae* and *Chlamydia pneumoniae* infections were common during acute asthma attacks [19]. Analysis of the microbiota in bronchoalveolar lavage (BAL), tracheal brushings, and sputum microorganisms revealed that the respiratory microbiota of asthmatic patients who had a significant increase in the abundance of *Proteobacteria* in the flora was significantly different from that of healthy people [20]. Through the cultivation of respiratory specimens (Figure 2), it was found that the proportions of asthma in children with bacteria, such as *Moraxella catarrhalis*, *Haemophilus influenzae*, or *Streptococcus pneumoniae*, were increased significantly, which were related to the severity and acute exacerbation of asthma [21, 22]. The results from 16S rRNA sequencing showed that the proportion of *Proteobacteria*, including *Haemophilus influenzae*, *Pseudomonas aeruginosa*, *Klebsiella pneumonia*, and other respiratory pathogens, in respiratory specimens of asthma patients was higher than that of the normal population. The increase in these pathogens was also found in patients with irregular inhaled hormone therapy, suggesting that this feature of respiratory flora was a characteristic of asthma itself and not simply a result of immunosuppression caused by inhaled hormones. In contrast, the proportion of *Prevotella* of *Bacteroidetes* in the airways of asthma patients was lower than that of the normal population [23–25].

The distribution of different bacterial communities is also related to the features of asthma. Airway microbes and asthma patients also have certain effects on the responsiveness of glucocorticoids. In vitro studies have shown that monocytes and macrophages cocultured with *Haemophilus influenzae* and *Haemophilus parainfluenza* were impaired in response to dexamethasone, which was found in some patients with hormone-resistant asthma [26]. Studies have shown that dexamethasone can inhibit tumor necrosis factor (TNF) production by macrophages and promote the phagocytosis of microparticles by human monocytes, thereby played an important role in immune-mediated tissue damage or tissue repair after infection [27, 28]. The possible mechanism of the effect of flora changes on the sensitivity to corticosteroids is the release of superantigens to produce oxidative stress, the release of cytokines, or the activation of host p38 mitogen-activated protein kinase (MAPK) [29].

These studies indicated that changes in respiratory flora would increase types of pathogenic bacteria but reduce types of normal flora and biomass in asthma patients, suggesting that the immune dysregulation of the respiratory flora may be one of the causes of asthma attacks.

## 4. Immunity of Respiratory Flora and COPD

COPD is a highly heterogeneous disease that seriously affects human health. It is characterized by persistent airflow obstruction and increasing airway chronic inflammatory response to harmful particles or gases. Exposure to noxious smoke and particles is a major driver for the progression of COPD. At the same time, other factors also play a part, such as genetic predisposition, nutritional status, and respiratory infection [30]. The role of microbial infection in the onset and development of COPD is a hot topic in the research of the respiratory microbiome. COPD is associated with the colonization of early pathogenic pathogens in the bronchus. The possible effect of changes in respiratory microbes on the progression of COPD can be explained by the circular vicious hypothesis [31]. Once the innate self-protective mechanism of the lung affected by smoke exposure and the airway microbial homeostasis is broken, pathogenic microbes can induce an inflammatory response of COPD, leading to progressive airway obstruction as well as pulmonary parenchyma injury by disrupting the balance of proteases and antiproteases in the lung. Direct supporting evidence for this hypothesis came from conventional biological research. A large increase in local immune and inflammatory components such as inflammatory corpuscles, cytokines, chemotactic factors, and proteases in the airways of patients with COPD could be detected by bronchoscopy or sputum examination. These pathological changes were consistent with chronic infection. Meanwhile, COPD patients with bronchiectasis harbored more airway bacteria, showing worse clinical outcomes [32]. Microbiological research can help us better understand the impact of microbes on the pathogenesis and progression of COPD. But whether differences in airway microbes have an impact on COPD clinical symptoms (such as the presence or absence of bronchitis) deserves further exploration.

Unlike the early changes in the microbiota of patients with asthma, there is no significant difference in respiratory flora between mild and moderate COPD patients and normal people [33]. Changes in flora are present only in patients with severe COPD (Forced expiratory volume in one second (FEV1) is less than 40% to 50% of the expected value). Microbiological analysis of sputum samples showed that the abundance of bacteria in the airway of patients with COPD was decreased, the proportion of *Proteobacteria* such as *Pseudomonadaceae*, *Burkholderiaceae*, and *Enterobacteriaceae* was increased, and the relative abundance of *Firmicutes* was decreased [34]. However, some scholars believe that *Firmicutes* and *Actinobacteria* were the dominant flora in the lungs of COPD patients. By sampling and sequencing the BALF of patients with moderate and severe COPD, Pragman et al. found that the structure of the flora in the lungs of these two groups of patients was changed and that *Firmicutes*, *Proteobacteria*, and *Actinobacteria* were abundant in the lower airways of these patients [35]. Microbiological analysis of bronchial biopsy showed that this change was related to the increase in local heterogeneity of respiratory microbes. This change in respiratory microbes associated with disease severity had also been reported in patients with CF [36].

Microbiological studies showed that, at the acute exacerbation stage of COPD, there was no significant difference in respiratory microbes compared with the stationary phase [37]. The respiratory symptoms in the acute exacerbation stage of COPD are related to the colonization of new strains in the airway. Microbiological analysis showed a clear increase in the relative abundance of bacteria in one genus but without significant changes in other colonies. In the process of infection, the microbial pattern was completely different [38]. The latter had an obvious increase in the proportion of *Firmicutes* before the colonization of the dominant bacteria. This finding also supported the clinical features of acute exacerbation in both types. Taking into account the different microbial modes, different therapeutic measures are required. Although the composition of respiratory flora in patients with COPD has been clear through microbiological analysis, the role of respiratory flora in the pathogenesis of the disease remains to be further explored.

## 5. Immunity of Respiratory Flora and CF

CF is a familial autosomal recessive inherited congenital disease, which is caused by mutations in the cystic fibrosis transmembrane conductance regulator (CFTR), resulting in the structural and functional alterations of the transmembrane protein in pulmonary CF, thereby causing chronic pulmonary infection and pulmonary function deterioration in patients, leading to early deaths [39]. CF is characterized by defective mucociliary clearance and chronic infection of complex microbial flora [40]. Infection, persistent inflammation, and acute periodic attacks lead to an irreversible decline in lung function. Although little is known about the factors contributing to acute exacerbation, antibiotic treatment can temporarily resolve lung symptoms and partially restore lung function. Acute exacerbations may be associated with changes in microbial density and the acquisition of new microbial strains.

Fodor et al. used massive pyrosequencing technology to identify changes in the airway microbial community structure of 23 CF patients during CF exacerbations and stabilities and obtained more than 350,000 sequences, representing nearly 170 distinct microbial floras [41]. Approximately 60% of the sequences obtained were from the known CF pathogenic bacteria *Pseudomonas aeruginosa* and *Burkholderiaceae*. Although antibiotic treatment and species richness declined slightly, the microbial community structure hardly changed. Moreover, the microbial composition in acute exacerbation was highly similar to that in the stationary phase, suggesting that exacerbations may be due to the spread of infection in the lungs, rather than changes in the composition of the microbial community.

The research through in vitro bacterial culture of the patients' sputa revealed that the predominant pathogenic bacteria were *Pseudomonas aeruginosa*, *Haemophilus influenzae*, and *Staphylococcus aureus*. Meanwhile, other strains such as *Burkholderia*, *Stenotrophomonas maltophilia*, and *Achromobacteria xyloxydans* were also present in elderly patients [42, 43]. The diversity of microbial communities in the lungs of CF patients was decreased, and the degree of pulmonary inflammation was related to the decrease in microbial diversity. Moreover, *Bacteroidetes* and *Fusobacteria* were the dominant strains in healthy individuals while *Actinobacteria* accounted for a larger proportion in CF patients. The structure of pulmonary florae was destroyed, pathogenic bacteria and conditional pathogens were multiplied, and florae were out of balance, which may be one of the causes of CF.

## 6. Immunity of Respiratory Flora and UACS

UACS, formerly known as postnasal drip syndrome (PNDS), is a syndrome of chronic cough caused by the reflux of allergic or nonallergic inflammatory secretions from the nasal cavity and nasopharynx into the pharynx [44]. It can be accompanied by a series of symptoms such as pharyngeal foreign body sensation, itchy pharynx, blocking sensation, and sputum adhesion in the pharynx. It is considered to be one of the most common causes of chronic cough. The pathogenesis of UACS remains unclear. Physicians in different countries have different definitions as well as treatments for UACS. The pathogenetic theories of UACS include the earliest theory of postnasal drip irrigation, the subsequent theory of chronic airway inflammation, and the more recent theory of sensory nerve hypersensitivity. Furthermore, some researchers believed that UACS was the clinical phenotype of irritable cough syndrome [45].

There is a slight difference in the types of pharyngeal flora between healthy people and patients with UACS. *Firmicutes* and *Proteobacteria* are the dominant flora in UACS that are different from the normal flora. Among these, *Lactobacillus* of *Firmicutes* and *Pseudomonas aeruginosa* of *Proteobacteria* have significantly increased and replaced the dominant position of original *Streptococcus* and *Neisseria* in the pharynx of normal people. Moreover, TRPV1 and TGF-*β*2 were increased significantly in UACS patients [46]. TRPV1 is a nonselective cation channel associated with cough sensitivity and widely distributed in sensory nerves from the upper to lower airways [47]. TRPV1 is regulated by multiple physicochemical factors, e.g., increased lactate secretion and the stimulation of inflammatory factors will lead to the increase of TRPV1. And it is highly expressed in pathological conditions such as chronic cough and high airway responsiveness. Another study showed significant elevation of both TGF-*β*1 and *β*2 in mammary secretions of dairy cows infected with *Pseudomonas aeruginosa* compared to those uninfected, demonstrating the correlation between *Pseudomonas aeruginosa* and TGF-*β*1 and *β*2. Meanwhile, *Pseudomonas aeruginosa* could also stimulate airway inflammation through a variety of known inflammatory pathways, leading to an increase in the prevalence of UACS [48].

## 7. Immunity of Respiratory Flora and Infection of Respiratory Fungal

Fungal colonization in the human body refers to the presence of a large number of fungi growing in the form of spores at a site where the human body communicates with the outside world, such as the alimentary canal, the upper airway, and urogenital tract, but without local tissues damage or symptoms [49]. Colonization is generally the final step in a long-lasting symbiotic or innocuous relationship between the fungus and the host and also is the first step in the conversion to fungal infection and the development of related diseases [50].

Contamination and colonization with *Candida* and *Aspergillus* in the upper airway are very common [51]. The results of ICU investigation in domestic general hospitals showed that *Candida* was the most common fungus colonizing in the lower airway, among which the infection rate of *Candida albicans* was the highest (37.91%), followed by *Aspergillus* (16.99%) [52]. Systemic or local immunosuppression caused by granulocytopenia in leukemia chemotherapy is an important cause of airway fungal infection. Pathogenic fungi can be colonized in patients, with infection and colonization by *Aspergillus fumigatus* and *Candida albicans* being the most common [53].

## 8. Inhaled Corticosteroids (ICSs) and Airway Flora Immunity

Glucocorticoid has a potent anti-inflammatory and immunosuppression function, which eliminates most pathogenic bacteria and improves the airway microenvironment by effectively reducing the levels of proinflammatory cytokines and airway inflammation. Inhaled corticosteroids, without obvious systemic side effects, can easily form an effective concentration in the airway and work directly, which is quickly destroyed by enzymes in alveoli and inactivated by the liver after entering the blood circulation.

Glucocorticoids can enter into the nucleus by binding with glucocorticoid receptor in the cytoplasm through the cell membrane and form hormone-hormone receptor complex, which combines with special DNA sequence to produce biological effect through the following three ways: (1) inhibiting the release of inflammatory factors such as TNF-*α* and leukotrienes by promoting the production of enzyme-linked protein I, resulting in the decrease of phospholipase A2-*α*; (2) inhibiting the production of inflammatory protein and its phospholipase A2-*α* by inactivating MAPK; (3) inhibiting the expression of cytokines, chemokines, cell adhesion factors, and their receptors by activating NF-*κ*B pathway [54]. Glucocorticoids not only can directly inhibit inflammatory cells but also reduce the exudation of capillary and the formation of sputum. Studies have found that dexamethasone reduced lung inflammation by promoting the secretion of IL-12 and inhibiting the expression of IL-13 in rats [55]. However, there are still side effects in the long-term repeated use of inhaled corticosteroids to control respiratory diseases, such as dryness, hoarseness, and *Candida* infection in the oral and pharyngeal [56]. Furthermore, long-term use of glucocorticoids may also cause immunosuppression and aggravate the development of the disease by destroying the local mucosal barrier and microenvironment of the airway, resulting in the unbalance of the microbial flora. These will lead to changes in the microbiome and increase the risk of some systemic reactions such as airway infections (*pneumonia* and *mycobacterial* disease). Thus, rationally controlling the time of using hormones may reduce the incidence of adverse reactions.

In addition, previous studies have proved that the diversity of oral microflora was decreased, while the proportion of *firmicutes* and *Bacteroidetes* was increased in normal obese children [57]. Because the diversity of flora is affected by many factors, maintaining the balance of flora is the main measure to reduce the occurrence of adverse reactions when infants are given Budesonide atomization therapy. Therefore, it is necessary to strengthen the detection of flora and maintain the balance of respiratory flora in clinical treatment to reduce the occurrence of adverse reactions [58].

Although ICSs have been widely used in the clinical treatment of respiratory diseases because of the strong anti-inflammatory activities, they could not effectively improve the inflammatory response of airway remodeling and proliferation. Most clinical reports have shown that excessive inhalation of ICSs may cause drug dependence or resistance and other adverse reactions, especially in children with asthma in the early stage, which will affect children's immune system and result in hormone-dependent. Therefore, exploring new anti-inflammatory drugs to combine or even replace hormones is the leading direction of drug development.

## 9. Probiotic Preparation and Improvement of Respiratory Flora Immunity

Probiotic refers to live biotherapeutic products (LBP) containing enough viable organisms with well-defined composition. It can change the composition of the flora at a certain site of the host through colonization, thereby improving the microecological balance of the host and playing a beneficial role [59]. On the one hand, probiotics can regulate the adaptive immune response of the host by promoting the release of cytokines from dendritic cells (DCs), altering the balance of Th1/Th2, shifting the immune response in the direction of Th1 cells, and inhibiting the response of Th2 cells. On the other hand, it can also play a role in the biological barrier by competing with pathogens and secreting antimicrobial peptides and other metabolites [60]. At present, probiotics have been used to treat diseases associated with immune dysregulation of the intestinal flora, such as diarrhea, obesity, type 2 diabetes, inflammatory bowel disease, and other diseases [61, 62]. In recent years, a probiotic composition has been invented, including *Lactobacillus rhamnosus* and *Lactobacillus plantarum*. This supplement inhibits the expression of inflammatory factors in the lung by activating TLR3 and RIG-1 signaling pathways in alveolar macrophages to promote IFN-*β* and also regulates lung flora and intestinal flora to alleviate respiratory syncytial virus (RSV) infection. Over-the-counter probiotics have been used in clinical trials to investigate their potential effects in various disease conditions, but it is required more stringent quality control to ensure the purity and efficacy of products. The challenge of detecting unwanted microbial contaminants is that the sensitivity of detection may be reduced in the presence of the required probiotic microorganisms. One of the strategies under study was to reduce or eliminate the growth of bacteria in the product for improving the sensitivity of detecting the contaminating microbes. FDA scientists developed and used recombinant phage lysine as a reagent to improve LBP purity detection through “mock” purity assays (“test-tube” studies) where *Lactobacillus jensenii* represented the probiotic's product strain. However, the type of strains, dosage, and course of probiotic can affect the outcome of clinical application, and even the same flora will have different effects at different ages. Therefore, the prevention and treatment of respiratory infection with probiotics deserves further research.

## 10. Protective Effects of Natural Products against Various Stimuli-Induced Lung Dysbiosis

Natural products or traditional Chinese medicine have been reported to improve airway immunity, ameliorate the function of the blood-air barrier, inhibit inflammation, and reduce growth of pathogenic bacteria due to its advantages of multicomponent, multichannel, and multitarget (Table 1), which had the potential for the prevention and treatment of several respiratory diseases including COPD, asthma, and UACS [63]. Baicalin, a kind of flavonoid derived from Scutellaria baicalensis (Huangqin in Chinese), with broad-spectrum antibacterial activity and obvious anti-inflammatory activity, which can effectively improve the respiratory system inflammation and treat respiratory disease by inhibiting a variety of bacteria and fungi [64, 65]. Baicalin has an inhibitory effect on *Candida albicans* and *Staphylococcus aureus*, which was correlated with the concentration of baicalin, that is, the DNA synthesis of *Candida albicans* was decreased with the increase of drug concentration [66, 67]. Cryptotanshinone has antiasthmatic effect, the mechanism of which may be related to downregulating Th2 cytokines and reducing inflammatory cell infiltration [68]. The Yinqiao powder is equipped with the effect of clearing away heat and toxin by inhibiting *Escherichia coli*, *Staphylococcus aureus*, and *Pseudomonas aeruginosa* [69]. Xiaochaihu decoction, a famous herbal medicine exerting antiallergic and antitussive effects, has been widely used for the treatment of asthma in clinics [70]. It can effectively reduce the frequency of asthma attacks and airway infection in children with asthma and alleviate airway inflammation by enhancing the immune function and antiallergic ability of airway mucosa. However, the current research is still insufficient. Most studies focus on in vitro antibacterial (*Streptococcus pneumoniae*, *Staphylococcus aureus*, etc.) activities of the agents, but few considered the antibacterial effect in vivo. And due to the complexity of pharmacodynamics, it is difficult to draw the conclusion whether antibacterial effects of drugs or anti-inflammatory effects play the decisive role in airway inflammation.

There is still a lack of robust research on pharmacological mechanisms. Therefore, further efforts are needed to elucidate the underlying mechanism in germ-free mice.

## 11. Conclusion

Changes in the immunity of respiratory flora play an important role in the development of multiple chronic respiratory diseases. In recent years, with the application of various new techniques in the detection of microbes in respiratory specimens, growing evidence showed that respiratory microbial population and its related local mucosal immunity were associated with the clinical manifestations, acute exacerbation, and prognosis of chronic respiratory immune disorders, such as asthma and COPD. Through studying the microbiological basis of the progression of human respiratory diseases, we can make a better understanding of the local immunological mechanism of the progression of these diseases, facilitate the judgment of disease types, predict the responsiveness to treatment, and evaluate the therapeutic effect. It provided a new idea for the clinical diagnosis, treatment, prediction, and prognosis of respiratory diseases.

## Figures and Tables

**Table 1:** The protective effects of traditional Chinese medicine on asthma.

Mechanism	Traditional Chinese medicine	Reference
Regulating Th1/Th2 balance	Bulleyaconitine A	[71]
	Ligustrazine	[72]
	Selaginella uncinata flavonoids	[67]
	Turmeric (Curcuma longa)	[73]

Regulating Th17/Treg balance	Bufei Yishen formula	[74, 75]
	Baicalin	[76]
	Gu-Ben-Fang-Xiao-Tang	[77]
	Louqin Zhisou decoction	[78]

Regulating antigen presenting cells	Atractylodin	[79]
	Oligomeric proanthocyanidins	[80]
	Tilianin	[81]
	Wuhu decoction	[82]

Inhibiting oxidative stress	Citrus flavonoids	[83]
	Platycodi Radix	[84]
	Quercetin	[85]
	Saikosaponin A	[86]
	Wedelolactone	[87]

Inhibiting NLRP3 inflammasome	Abscisic acid	[88]
	Andrographolide	[89]
	EGCG	[90]

## Data Availability

The data used to support the findings of this study is included within the article.
